# High Impact: The Role of Promiscuous Binding Sites in Polypharmacology

**DOI:** 10.3390/molecules24142529

**Published:** 2019-07-10

**Authors:** Natacha Cerisier, Michel Petitjean, Leslie Regad, Quentin Bayard, Manon Réau, Anne Badel, Anne-Claude Camproux

**Affiliations:** 1 Université de Paris, Biologie Fonctionnelle et Adaptative, UMR 8251, CNRS, ERL U1133, INSERM, Computational Modeling of Protein Ligand Interactions, F-75013 Paris, France; 2 Centre de Recherche des Cordeliers, Sorbonne Universités, INSERM, USPC, Université Paris Descartes, Université Paris Diderot, Université Paris 13, Functional Genomics of Solid Tumors Laboratory, F-75006 Paris, France; 3 Laboratoire Génomique Bioinformatique et Chimie Moléculaire, EA 7528, Conservatoire National des Arts et Métiers, F-75003 Paris, France

**Keywords:** proteochemometrics, multi-targets, drug promiscuity, druggable binding site, descriptors, polypharmacology

## Abstract

The literature focuses on drug promiscuity, which is a drug’s ability to bind to several targets, because it plays an essential role in polypharmacology. However, little work has been completed regarding binding site promiscuity, even though its properties are now recognized among the key factors that impact drug promiscuity. Here, we quantified and characterized the promiscuity of druggable binding sites from protein-ligand complexes in the high quality Mother Of All Databases while using statistical methods. Most of the sites (80%) exhibited promiscuity, irrespective of the protein class. Nearly half were highly promiscuous and able to interact with various types of ligands. The corresponding pockets were rather large and hydrophobic, with high sulfur atom and aliphatic residue frequencies, but few side chain atoms. Consequently, their interacting ligands can be large, rigid, and weakly hydrophilic. The selective sites that interacted with one ligand type presented less favorable pocket properties for establishing ligand contacts. Thus, their ligands were highly adaptable, small, and hydrophilic. In the dataset, the promiscuity of the site rather than the drug mainly explains the multiple interactions between the drug and target, as most ligand types are dedicated to one site. This underlines the essential contribution of binding site promiscuity to drug promiscuity between different protein classes.

Academic Editor: J.B. Brown

## 1. Introduction

Since the late-1960s, drug development processes have been based on the “one drug, one target” paradigm. The primary goal of drug discovery has been the design and delivery of selective compounds against individual biological targets [1]. A drug may be involved in different disease functions and might interact with more than one target, which is defined as drug promiscuity [1,2,3]. These multi-target drugs are commonly referred to as promiscuous drugs and they are the origin of polypharmacology, where drugs bind to more than one protein target at concentrations that are relevant to their therapeutic free exposure [4]. Drug promiscuity is accepted as a general phenomenon, with the growing number of compounds associated with multiple target annotations [5]. Jalencas et al. [6] reported that only 15% of drugs are known to interact with a single target, but over 50% interact with more than five targets; thus, drug promiscuity is a key element in drug discovery and development. Its consequences can be either beneficial or undesirable. Amongst the beneficial outcomes, the drug may be applicable against new diseases, avoiding time and money being spent on preclinical tests or repurposing [7,8]. Amongst the undesirable outcomes, promiscuous drugs can interact with off-targets, which results in adverse drug reactions, harmful side effects, and adverse polypharmacology [1,7,9].

Consequently, drug promiscuity has received considerable attention. It has been analyzed while using the drug physicochemical properties, fragment composition, the protein family to which they bind, and the binding site similarities by taking advantage of the increasing number of three-dimensional structures in a complexed form. The protein binding site similarity confers similar binding interactions with a common ligand. For instance, Haupt et al. [2] demonstrated that the global structure and the binding site similarity within the structures of the Protein Data Bank (PDB) [10] had greater influence on drug promiscuity than physicochemical drug properties, such as hydrophobicity, molecular weight, or flexibility.

The field of binding site comparison has emerged as a powerful approach for investigating drug promiscuity. Naderi et al. [11] surveyed 12 widely used computational tools to match the pockets and underlined the important pharmacological applications of pocket matching. For related targets, small molecules often bind with a high affinity to multiple members of a protein family due to the high sequence similarity in its active sites [2,12]. Duran-Frigola et al. [13] and Meyers et al. [14] confirmed that binding site pocket similarity analysis is a useful tool in promiscuity detection within the same family, being named intrafamily promiscuity. However, the performance of the binding site comparison has not been demonstrated concerning interfamily promiscuity detection, when the promiscuous drug acts across different target families. Interfamily promiscuity is rarely observed; only ~2% of bioactive compounds are considered to be promiscuous across different unique target families on the basis of high-confidence activity data [15]. Their importance should not be neglected for drug repurposing, the prediction of side effects and drug–target interactions, leading optimization, and prioritizing the study of secondary targets for their importance in efficacy or toxicity [14,16]. Similar pockets may occur within unrelated protein structures [17] and one-third of similar cavities from experimental human protein structures can be found among the proteins with no apparent relationship, as related in [13]. They identified 181,500 pairs of similar cavities: 68.8% corresponded to pockets in different structural instances of the same protein and 31.2% to cavities in distinct proteins. High-affinity selective interactions can occur for a ligand when binding to different protein families, despite the absence of an apparent binding site or sequence similarity [18,19]. An analysis by Barelier et al. [20] concerning ligands that are able to bind proteins from different families showed that the majority of examples of binding sites in unrelated proteins were not a result of matched residues in the ligand binding site. They concluded that there was no single pattern-matching “code” to identify the binding sites that bound identical ligands in unrelated proteins. Meyers et al. [14] concluded that the pocket similarity analysis performance for the promiscuity detection has not been demonstrated to be relevant when searching for proteins outside the family of the target protein in some chemotype cases. They highlighted the importance of understanding the factors that control the underlying similarity in protein binding sites. Numerous studies have confirmed that the plasticity of the binding site is important in protein–ligand interactions [21,22,23]. Gao et al. [24] concluded that more than one-third of their representative pockets from the PDB were promiscuous and they interacted with multiple different ligands. Hu et al. [25] analyzed both the compound and target promiscuity and the target’s ability to interact with an increasing number of structurally diverse compounds and concluded that most of the targets bind to varying numbers of promiscuous compounds.

To the best of our knowledge, no exhaustive work has been dedicated to quantifying and characterizing the structural features of target binding sites to understand their propensity to be promiscuous versus selective (that is, able to interact with only one type of ligand). It is possible to explore the target binding site space and multiple binding site–ligand interactions while taking advantage of the increasing number of three-dimensional (3D) structures and their redundancy. In this study, we used the interaction database, Mother Of All Databases (MOAD), which is one of the largest databases that provides more than 25,000 protein–ligand complexes with only high-quality resolution structures extracted from the PDB that are suitable for studying the promiscuity of binding sites [26]. As druggability is crucial in drug design [4,27], we focused on druggable binding sites (DBS). A DBS is defined by its corresponding pocket set estimated by proximity to a drug-like ligand from homologous PDB chain clusters (chain with 90% sequence identity) in the MOAD. Here, we investigated the promiscuity of DBS that were observed several times in the MOAD (at least four pockets) and their frequency. Next, we studied the characteristics of pockets and ligands corresponding to the DBS of different promiscuity in terms of their geometrical and physicochemical properties and their correspondence. Subsequently, we studied the DBS promiscuity frequency within different MOAD protein classes and their contribution to explain the drug promiscuity between the MOAD protein classes. A network study allowed for the visualization of the multiple connections between DBS and different ligands and confirmed the high impact of DBS promiscuity on these multiple connections. This work demonstrates the importance of DBS promiscuity analysis for understanding and modeling multiple protein–ligand interactions, drug promiscuity, polypharmacology, drug repositioning, or off-target detection.

## 2. Results

### 2.1. MOAD Druggable Binding Site Identification

#### 2.1.1. MOAD Protein and Ligand Space

**Figure 1:**
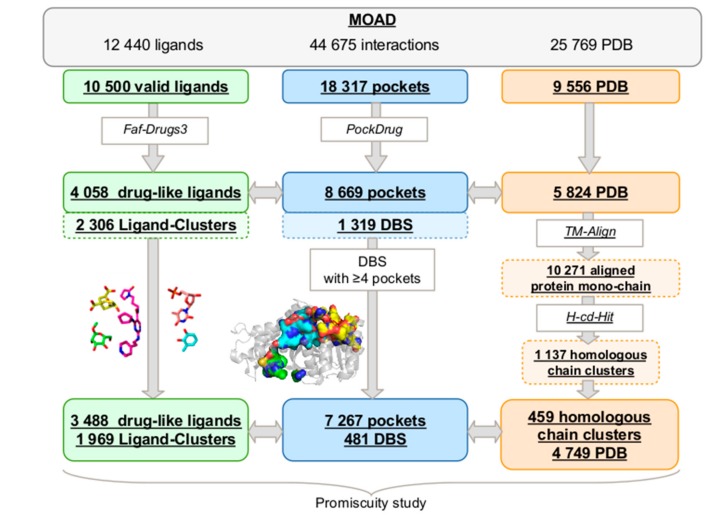
The protocol developed to extract Druggable Binding Sites (DBS) from the Mother Of All Databases (MOAD) data and to determine their promiscuity. We extracted 3,488 valid and drug-like ligands that were regrouped into 1,969 clusters while using the Tanimoto coefficient (green), 459 homologous chain clusters among the 4,749 Protein Data Bank (PDB) files selected using TM-Align [28] to calculate the identity between all the protein chains, and H-CD-HIT [29] clustering algorithms to group the homologous protein chains (orange). The 7,267 pockets clustered in 481 druggable binding sites (DBS) (blue) correspond to the ligands previously selected and all of the homologous protein chains. The promiscuity was calculated from these data.

The MOAD provides 25,769 high-resolution protein–ligand complexed structures, which include 12,440 unique ligands and a total of 44,675 protein–ligand interactions. First, we extracted 10,500 different valid ligands (see Section 4) from 9,556 PDB complex structures that were involved in 18,317 pocket–ligand interactions (Figure 1). Selected molecules were filtered based on their drug-likeliness, and 5,824 PDB complex structures containing 4,058 valid and drug-like ligands were retained. From these PDB files, we aligned 10,271 protein mono-chains while using TM-Align, clustering them into 1,137 homologous chain clusters using H-CD-HIT. The final filtered dataset included 8,669 protein–ligand interactions.

Next, similar ligands were clustered while using a Tanimoto coefficient threshold of 0.8 in the “Ligand–Cluster”. The 4,058 ligands were clustered into 2,306 Ligand–Clusters, which indicated that more than half of the ligands (56.8%) were diverse. The average Tanimoto coefficient between the pairs of representative ligands from all Ligand–Clusters (see Section 4) was weak at 0.32 (±0.13), confirming that the MOAD includes large valid and drug-like ligand diversity. On average, these 2,306 Ligand–Clusters included 1.75 (±2.23) similar ligands. Most of them (71%) were only observed once in the MOAD, but some were also highly observed in the MOAD. For example, the largest Ligand–Cluster included 46 similar ligands (meaning different Het molecules as defined in the PDB but with a Tanimoto > 0.8) that corresponded to a derivate of nucleosides.

Thus, the MOAD provides a number of proteins, ligands, and protein–ligand interactions that are sufficiently diverse to study the promiscuity of DBS obtained from homologous chain clusters in different MOAD protein classes.

#### 2.1.2. Druggable Binding Site Extraction 

From the selected protein–ligand interactions, 8,669 binding pockets were estimated by proximity to the 4,058 drug-like ligands while using a threshold of 5.5 Å (Figure 1). We applied our Pocket-Clustering algorithm to these pockets to identify those describing an identical druggable binding site (see Section 4). It located 8,669 binding pockets from the 1,137 different homologous chain clusters and clustered them into 1,319 sets of overlapping pockets, named the “Pocket-Cluster”, which described 1,319 distinct DBS. These 1,319 DBS are described, on average, by 6.57 (±13.43) pockets, with a minimum of 1 and a maximum of 164 pockets per DBS. The average score of overlap within the Pocket-Cluster was 0.65 (±0.13), with a minimum of 0.25 and a maximum at 0.95. This minimum confirmed that our Pocket-Clustering algorithm split the non-overlapping pockets that corresponded to disconnected regions of a binding site in a different Pocket-Cluster, which is coherent for studying the DBS promiscuity.

To determine the promiscuity of a DBS, we had to quantify its number of different Ligand–Clusters in interaction with its Pocket-Cluster. We defined a DBS in interaction with only one Ligand–Cluster as a “selective DBS”. A qualification of DBS selectivity can be due to the limited availability of three-dimensional (3D) crystal structures complexed in the MOAD. One-third of obtained DBS (35%) were observed only once in the MOAD (and described by a unique binding pocket); consequently, they cannot be technically characterized in terms of promiscuity. The frequency of selective DBS directly decreased with DBS occurrence in the MOAD (results not shown). Thus, the higher the occurrence of a DBS in the MOAD, the higher the possibility of observing its interactions with different Ligand–Clusters, and the higher the reliability in its qualification of promiscuous or selective DBS. We selected a compromise between having enough occurrences of each DBS (important enough to limit the characterization of DBS as “selective” when not true) to analyze the promiscuity of each DBS, but also to retain a high number of different DBS to obtain reliable promiscuity information from the MOAD. In this study, we chose to analyze the promiscuity of the set of 481 DBS observed four or more times, which is referred to as the DBS4 dataset (the complete dataset is available at the following DOI: 10.6084/m9.figshare.8313185). These corresponded to 37% of the DBS that were described by 7,267 pocket–ligand interactions from 459 homologous chain clusters and a large part of the binding pockets (83%) in interactions with 86% of the ligands. These 481 DBS interacted with 3,488 ligands, which were clustered into 1,969 Ligand–Clusters. These DBS are described by 15.1 (±19.5) pockets, on average, with a Pocket-Cluster size between four and 164 pockets and interacted with 8.0 (±14.0) ligands. 

### 2.2. DBS Promiscuity Characterization

#### 2.2.1. Druggable Binding Site Promiscuity Quantification 

**Figure 2:**
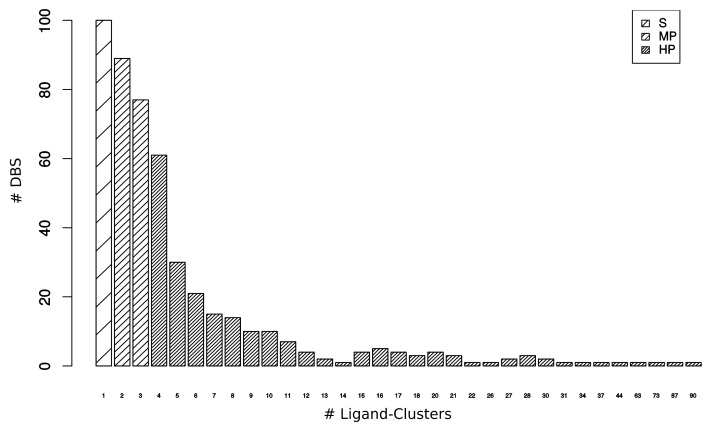
Number of DBS interacting with 1 to 90 Ligand–Clusters from the DBS4 dataset. Among the 481 DBS, 100 were selective (S), 166 were moderately promiscuous (MP), and 215 were highly promiscuous (HP).

Complementary to selective DBS, we defined a DBS interacting with several Ligand–Clusters as a “promiscuous DBS”. We quantified the DBS interacting with only one type of ligand versus several types of ligands in the DBS4 dataset to determine the promiscuity of the DBS. The DBS was associated with 5.6 (±8.9) Ligand–Clusters on average, from 1 to 90 Ligand–Clusters (Figure 2). Almost one-quarter of the DBS (21%, 100/481) were selective, whereas 79% were promiscuous DBS (Table 1). Amongst the promiscuous DBS, we distinguished 34% (166) as moderately promiscuous (MP) DBS, which were associated with two or three Ligand–Clusters, from 45% (215) highly promiscuous (HP) DBS, which were associated with four or more Ligand–Clusters. The most promiscuous DBS corresponded to the Human Cyclin-dependent kinase 2 (CDK2) transferase, which has been well studied due to its role in the regulation of the acid–base balance in the organism [30]. Its high pocket (164) and Ligand–Cluster (90) numbers were consistent with the high occurrence of complexed structures, the protein high sequence homology of CDK families, and the numerous inhibitors that were developed against CDK2 [31].

Eight and seven pockets, on average, describe Selective and MP DBS, respectively, whereas 23 pockets describe HP DBS. The 45% HP DBS corresponded to 69% (5,029) of the 7,267 pockets (Table 1). However, it was difficult to interpret whether these high pocket occurrences of HP DBS are due to these DBS that belong to targets of therapeutic interest and they are highly crystallized (which contributes to revealing their high promiscuity), or a consequence of their known promiscuity, which explains that they have been intensively studied and crystallized with different ligands. As expected, the 100 selective DBS that interacted with only one Ligand–Cluster each corresponded to a small part of the Ligand–Clusters (less than 4%). The 166 MP DBS interacted with 15.3% of the Ligand–Clusters. The 215 HP DBS corresponded to 89% of the Ligand–Clusters (and 5,029 pockets), according to their high ability to interact with various types of ligands, which resulted in 7.31 Ligand–Clusters on average per DBS. Thus, we conclude that DBS promiscuity is a common phenomenon in proteins.

#### 2.2.2. Binding Pocket Characteristics of DBS with Different Promiscuity Levels

**Figure 3:**
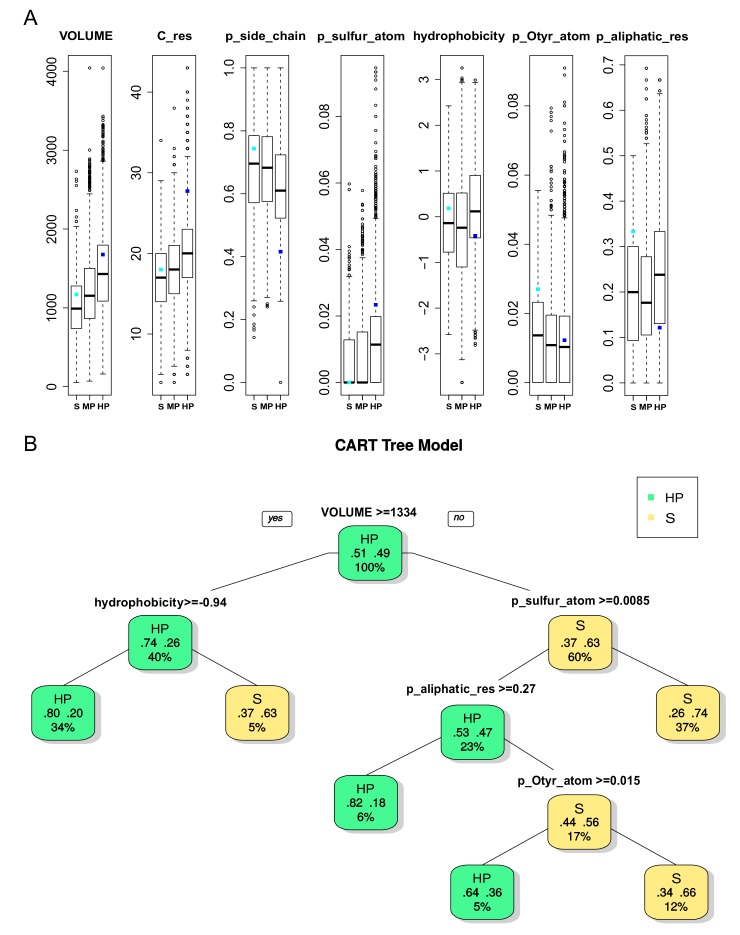
(A) Boxplot of seven pocket properties that differentiated DBS with different levels of promiscuity (p value < 2×10–5). For each boxplot, the black bar in the rectangle represents the medium, the upper side is the third quartile, and the lower side is the first quartile of data. The dashed lines represent values below the first quartile limit and values above the third quartile limit. Circles represent atypical values (determined by the boxplot function). The cyan dot and the blue dot represent the values of the ligand in interaction with a lyase selective DBS (PDB 4KVI) and an urokinase HP DBS (PDB 1F5K), respectively, as illustrated in Figure 5. (B) Representation of the Classification And Regression Tree (CART) for pockets based on the pocket descriptors between the S (yellow) and HP (green) pockets. Each node is described by the class (promiscuity), the probability per class (right = HP and left = S), and the percentage of observations in the node. The CART model performances obtained for distinguishing pockets associated with the selective DBS from the HP DBS resulted in an average accuracy, sensitivity, and selectivity of 68%, 75%, and 67% for the test set (1,252 pockets), respectively, and 64% ±2%, 60% ±5%, and 64% ±3% while using a five-fold cross validation on the training set (500 runs), respectively.

We studied the physicochemical and geometrical pocket properties that discriminated the DBS of the three different levels of promiscuity. To do so, 72 physicochemical and geometrical pocket descriptors described each pocket. First, the results of ANOVA tests when comparing the pocket descriptors that were associated with the DBS of different promiscuity levels showed many significant differences (68/72 descriptors, Table S1). The most different descriptors corresponded to geometry, frequency of certain atoms, or residues and hydrophobicity. Some of them (with a *p* value < 2 × 10^−5^) are illustrated on a boxplot (Figure 3A). In terms of geometrical descriptors, the volume and number of residues increased with DBS promiscuity, which indicated that selective DBS correspond to rather small pockets than the HP DBS pockets (16.6 ±4.3 versus 20.1 ±5.1 in the number of residues). Selective DBS also corresponded to significantly smaller Pocket Convexity Index (PCI) values (Table S1), which thus supports more convex pockets. In terms of physicochemical descriptors, the proportion of side chain atoms was noticeably weaker and the proportion of sulfur atoms was significantly higher for pockets that are associated with HP relative to selective DBS. Sulfur atoms are known to form hydrogen bonds that are essential in many enzymatic reactions and they should contribute to DBS promiscuity [32]. The HP DBS pockets exhibited higher values of hydrophobicity (based on the hydrophobicity property of residue) for HP than the selective or MP DBS pockets. The frequency of aliphatic residues increased, while the frequency of oxygen atoms in tyrosine residue atoms (Otyr) decreased for the HP DBS pockets. The MP DBS pockets tended to present intermediate values between the selective and HP DBS pockets, except in some descriptors, such as the high value of frequency of nitrogen atoms in tryptophan residue atoms (Ntrp) and charged residues (Table S1). Pockets were associated with a high average druggability score in terms of the druggability score (78% ±0.26), which was in agreement with the drug-like ligand selection. The HP DBS tended to be the most druggable (81%), which agrees with the druggability score increasing with the size and hydrophobicity of the pockets [33]. The relatively small size of the selective pockets appeared to be compensated for by physicochemical properties, such as high Otyr atom frequency (which characterizes a hydrogen bond donor group by a hydroxyl group in the tyrosine side chain), which plays a key role in binding the drug-like molecules to explain their druggability score [34].

Second, we illustrated the pocket properties that discriminated the two most informative classes of DBS promiscuity: selective and HP DBS, while using the Cart And Regression Tree (CART) method. The CART model performance for distinguishing pockets that are associated with selective from HP DBS resulted in an average accuracy, sensitivity, and selectivity of 68%, 75%, and 67%, respectively, for the test set. Figure 3B presents a tree that illustrates the optimal combinations of pocket descriptors that discriminate HP from selective DBS, where different combinations explain different DBS promiscuity. Aligned with the ANOVA results, the first discriminating pocket property was the volume, and then different physicochemical properties were involved. HP DBS mainly corresponded to the largest pockets, except for those that were weakly hydrophobic. However, a few small pockets with high sulfur, aliphatic, or Otyr atom frequency were associated with HP DBS. Conversely, selective DBS were mainly associated with small pockets (78.6%) with weak sulfur atom frequency; otherwise, they had weak aliphatic residue and Otyr atom frequency.

#### 2.2.3. Ligand Characteristics interacting with DBS with Different Promiscuity Levels

**Figure 4:**
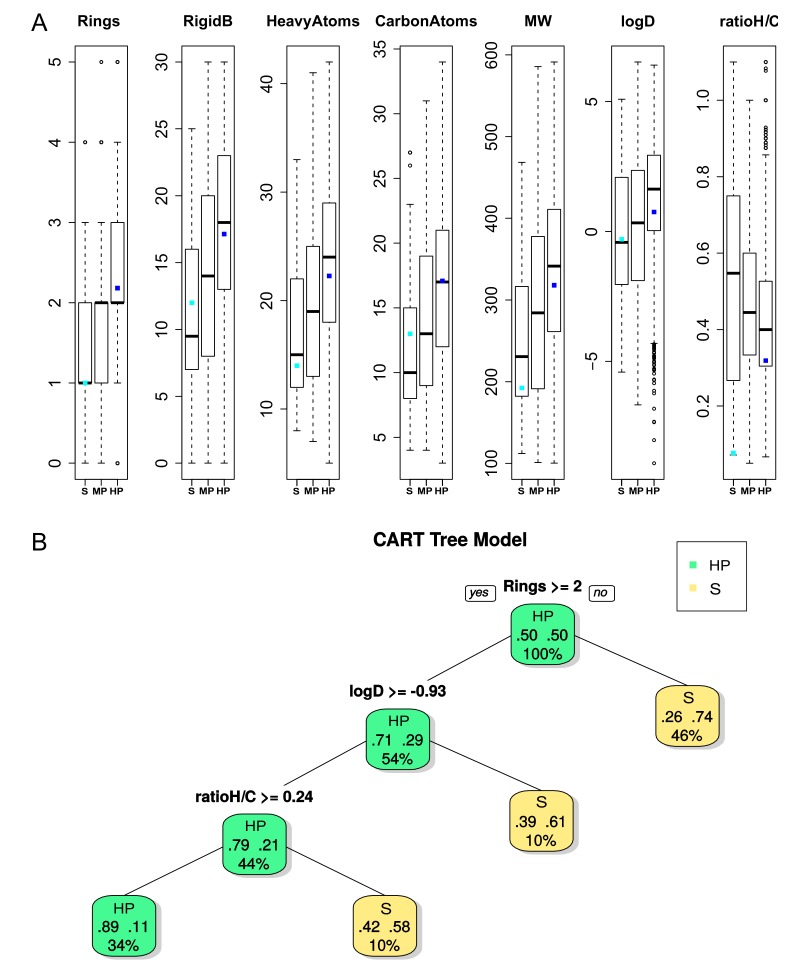
(**A**) Boxplot of seven ligand properties (*p*-value < 3×10^−10^ except for the ratio between hydrogen and carbon atoms called ratioH/C, *p*-value = 0.1) from ligands associated with the Ligand–Cluster dedicated to S or HP DBS. For each boxplot, the black bar in the rectangle represents the medium, and the upper side is the third quartile and the lower side is the first quartile of data. The dashed lines represent values below the first quartile limit and values above the third quartile limit. Circles represent atypical values; the cyan dot and the blue dot represent the values of the ligand in interaction with a lyase selective DBS (PDB 4KVI) and an Urokinase HP DBS (PDB 1F5K), respectively, as illustrated in Figure 5. (**B**) Representation of the CART tree for ligands based on ligand descriptors between S (yellow) and HP (green) ligands. Each node is described by the class (promiscuity), the probability per class (right = HP and left = S), and the percentage of observations in the node. The CART model provided an average accuracy, sensitivity, and specificity of 77%, 67%, and 77% for the test set (53 versus 2,621 ligands), respectively, and 72% ±3%, 69% ±7%, and 73% ±3% while using a five-fold cross validation on the training set (500 runs), respectively.

Of the Ligand–Clusters, 93.6% were associated with DBS presenting the same promiscuity level. Most of them (82.8%) only interacted with HP DBS according to the ability of these DBS to interact with a high number of Ligand–Clusters. We observed that these Ligand–Clusters interacting with DBS of the same level of promiscuity exhibited high diversity in terms of the Tanimoto coefficient (0.33), with the lowest Tanimoto value (0.24) for those that only interacted with selective DBS (Table 2). The ability of these Ligand–Clusters to interact with only the DBS of the same promiscuity level suggests that their ligand properties could correspond to the DBS pocket tendencies of corresponding promiscuity level. First, we characterized the ligands that were only clustered within these 93.6% Ligand–Clusters dedicated to selective or MP or HP DBS. The results of the ANOVA tests showed significant differences between all 21 ligand descriptors (Table S2), where seven are illustrated on a boxplot in Figure 4A. The ligand ability to bind to selective DBS decreased with its number of rings (Rings) and rigid bonds (RigidB) values, the carbon and heavy atom frequency, the molecular weight, and the lipophilicity values. Ligands that only interact with selective DBS were more flexible, as they exhibited a lower number of rings and RigidB values (the more important the number of rings and the value of RigidB, the less flexible the molecule). They tended to be lower in molecular weight, with less carbon and heavy atoms, and exhibited a lower value of lipophilicity (logD and logP), which means that they are more hydrophilic. The ratio between hydrogen and carbon atoms (ratioH/C) was particularly variable for selective DBS ligands, but it tended to be small for HP DBS ligands. Ligands that were bound to MP DBS mainly presented intermediate values between those that were bound to selective or HP DBS. Second, we studied the ligand properties that discriminated the Ligand–Clusters interacting with selective and HP DBS while using the CART model. The CART model performance produced an average accuracy, sensitivity, and specificity of 77%, 67%, and 77% for the test set, respectively. The tree that was obtained for the 53 ligands from the Ligand–Clusters interacting with selective DBS and the 2,621 ligands from those interacting with HP DBS is illustrated in Figure 4B, which confirmed that flexibility is the most important property for discriminating ligands that bind to HP versus selective DBS. Ligands with few rings or with low lipophilicity, or with a low ratio between the hydrogen and carbon atom values, mainly interacted with selective DBS. Thus, we conclude that ligands from Ligand–Clusters that were dedicated to selective DBS tend to be smaller, less rigid, with less carbon and heavy atoms, and tend to be more hydrophilic. This is coherent with their interaction with pockets that are difficult to bind (given their physicochemical and geometrical properties).

Only 6.6% of the 1,969 Ligand–Clusters interacted with DBS of different promiscuity levels. These we called the “mixed” Ligand–Clusters, and they corresponded to 16% of the 3,488 ligands (Table 2). They were split into 39 (2%) Ligand–Clusters interacting with 39 both selective and some promiscuous DBS, and 88 (4.4%) interacted with both MP and HP DBS. ANOVA tests between 2,931 ligands from the dedicated Ligand–Clusters versus 557 ligands from the mixed Ligand–Clusters showed that ligands were able to interact with DBS presenting different promiscuity (mixed Ligand–Clusters) and they tended to be more hydrophilic (low logP values) with a low number of rotatable bonds (RototableB) (Table S3).

#### 2.2.4. Pocket and Ligand Property Correspondence

We observed significant physicochemical and geometrical tendencies for both pockets and ligands that are associated with DBS of different promiscuity and therefore studied their correspondences. Both the pockets and ligands from selective DBS were significantly smaller than those that were associated with HP DBS. This pocket–ligand size correspondence was expected, as the pocket was estimated by its proximity to its ligand.

**Figure 5:**
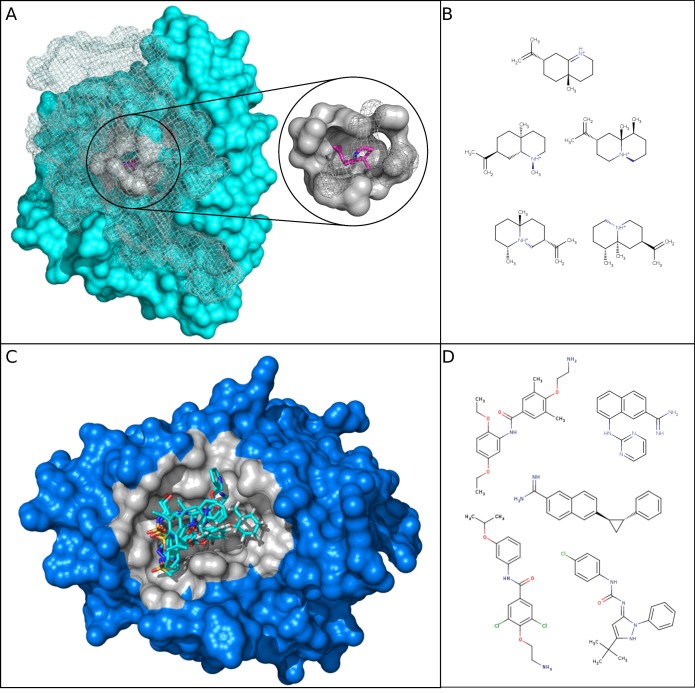
Illustration of one selective and one HP DBS and some of their associated ligands. (**A**) The aristolochene synthase protein with a selective DBS is represented in cyan with its representative structure: a lyase from *Aspergillus terreus* (PDB 4KVI, Pocket-Cluster no. 141_1). The grey part (in the surface and mesh) is the pocket that interacts with the ligand, where the carbon is represented by pink and the nitrogen in blue (PDB 1SV, Ligand–Cluster 300). Part of the protein and pocket are represented in mesh instead of the surface to facilitate the visualization of the pocket buried in the three-dimensional (3D) protein. (**B**) Five ligand two-dimensional (2D) structures (Tanimoto > 0.8) from the Ligand–Cluster associated with the selective DBS. (**C**) The human urokinase plasminogen activator protein with a HP DBS is represented in blue with its representative structure: a hydrolase from *Homo sapiens* (PDB 1F5K, Pocket-Cluster no. 94_2). Its pockets are represented in grey and five representatives of the 17 different Ligand–Clusters that can bind to the DBS (carbon in cyan, nitrogen in blue, oxygen in red, and sulfur in orange). (**D**) Five ligand structures in two-dimensional (2D) (Tanimoto < 0.8) among the 17 different Ligand–Clusters that can bind to the HP DBS. Structures were visualized using PyMOL [37].

Most selective DBS corresponded to small pockets with a high side chain atom frequency, a low proportion of sulfur, Otyr atoms, and aliphatic residues. A few of them were larger, but they presented low hydrophobicity values. These pocket properties are less favorable for establishing contact with various ligands. Accordingly, to be able to bind to these difficult pockets, their interacting ligands tend to be flexible and small with low carbon and heavy atom proportions and they are hydrophilic. Figure 5A illustrates one selective DBS, the aristolochene synthase from *Aspergillus terreus* from the lyase protein class. It is associated with 19 structure chains in the MOAD and five different ligands (clustered into only one Ligand–Cluster and corresponding to a Tanimoto coefficient of 0.88 ±0.07). As shown in Figure 3A and Figure 4A, its properties matched well with the selective tendencies for both the pocket and ligands.

The HP DBS corresponded to pockets that were well adapted to bind several and diverse ligands—these were mainly large and hydrophobic. For relatively small pockets, they presented high frequencies of sulfur atoms and aliphatic residue (or Otyr atoms), but low side chain atom frequencies. These HP DBS pocket properties are favorable for establishing interaction with different ligands. Therefore, their interacting ligands do not need a particular flexibility and adaptability to bind. Accordingly, we observed that their ligands tended to be large, rigid, and have high carbon and heavy atom frequencies. They were also less hydrophilic than those that are associated with selective DBS. Figure 5C illustrates one HP DBS, which is the catalytic inhibitor-binding site from the human urokinase Plasminogen Activator target from the hydrolase protein class. This target has been extensively studied due to its role in cancer pathways and for its high promiscuity [35,36]. The corresponding urokinase DBS presented pocket and ligand property values that matched well with the HP tendencies, as shown in Figure 3A, Figure 4A. The 22 corresponding pockets in the MOAD were large and hydrophobic, with a small proportion of side chain atoms and a high proportion of sulfur atoms. The ligands from its 17 associated Ligand–Clusters had high rigid bonds values, meaning that they were more rigid. 

### 2.3. DBS Promiscuity Contribution to Multiple Interactions of Ligands with Different MOAD Protein Classes

#### 2.3.1. DBS Promiscuity Frequency Related to MOAD Protein Classes 

Another question of interest was: “Can DBS of different promiscuous levels be observed within all MOAD protein classes or are they dependent on the protein class?” 

**Figure 6:**
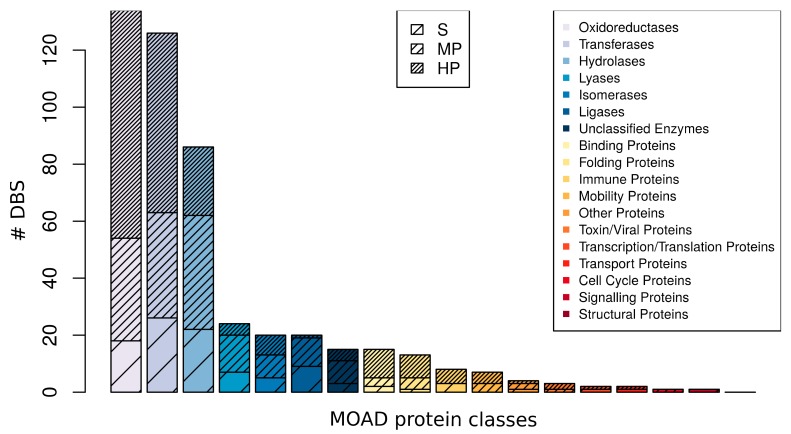
Distribution of the occurrence of DBS observed in the MOAD protein classes and their level of promiscuity in the DBS4 dataset. For instance, the lyase family corresponded to 20 DBS, with 5 S, 8 MP, and 7 HP DBS.

We observed that the 481 DBS were distributed among the 17 MOAD protein classes (Figure 6). The three most frequently observed protein classes were the enzymes transferase, hydrolase, and oxidoreductase, which together composed 71.9% of the DBS. We observed a high promiscuity with 4.64 (±2.49) Ligand–Clusters per DBS, on average, per protein class. For instance, there were 1.2 Ligand–Clusters per DBS on average for the binding protein class and 5.9 for the lyase protein class. We observed different promiscuous levels of DBS within different MOAD protein classes, independent of their frequency. The three promiscuous levels of DBS were observed in the nine most observed protein classes. Selective DBS and HP DBS were observed in the 10 and 15 most frequent MOAD protein classes, respectively. Moreover, there is a wide diversity of Ligand–Clusters that are associated with each HP DBS of different MOAD protein classes. The Tanimoto coefficient of Ligand–Clusters that was associated with the HP DBS of different MOAD protein classes was, on average, 0.44 (±0.10) per DBS per protein class, with a minimum value of 0.34 (±0.11) for the oxydoreductase and a maximum of 0.52 (±0.09) for the isomerase HP DBS (Table S4). This confirms that DBS with different promiscuous levels can be observed in diverse protein classes with the majority of HP DBS that interact with diverse ligands.

#### 2.3.2. Complementary Study of the Ligand–Cluster Promiscuity

Subsequently, we studied the contribution of the DBS promiscuity and the Ligand–Cluster promiscuity on the multiple interactions between the different protein classes. First, we conducted a complementary study of the promiscuity of the Ligand–Clusters in the DBS4 dataset. We observed a limited Ligand–Cluster promiscuity: 82.2% were selective (in interaction with only one DBS) and 17.8% were promiscuous. This weak rate of promiscuous Ligand–Clusters can be partly explained by 71% (1,407/1,969) of the Ligand–Clusters being composed of only one ligand (where 59% (832/1,407) only had one occurrence) in the DBS4 dataset. These promiscuous Ligand–Clusters were associated, on average, with 3.11 (±2.54) DBS extracted from 1.76 (±1.02) different MOAD protein classes.

We observed that most of the Ligand–Clusters were associated with DBS of the same promiscuous level. Thus, we studied the frequency of promiscuity of Ligand–Clusters relative to that of their interacting DBS. Only 18.9% the 1,753 Ligand–Clusters in interaction with at least one HP DBS were promiscuous; these interacted with 3.15 DBS, on average, corresponding to 1.76 MOAD protein classes. This could be due to HP DBS pocket properties being favorable for establishing interaction with different ligands and, consequently, their interacting ligands do not need to be particularly flexible, which does not support their ability to interact with several DBS. A total of 44.2% and 57.3% of Ligand–Clusters interacting with at least one MP DBS and at least one selective DBS were promiscuous, respectively (Table 3). This is coherent with their tendency to be flexible and small for interacting with more difficult pockets that are associated with selective DBS. These promiscuous Ligand–Clusters interacted with a high number of DBS: 4.15 DBS corresponding to 2.13 MOAD protein classes and 6.71 DBS corresponding to 2.95 MOAD protein classes, respectively. 

Next, we analyzed the 68 Ligand–Clusters that interacted with selective DBS of different MOAD protein classes in further detail. These were split in Ligand–Clusters different in terms of promiscuity. We studied the characteristics and frequency of the 29 selective and the 39 promiscuous Ligand–Clusters. The 29 selective Ligand–Clusters interacted with 29 selective DBS that belonged to nine different MOAD protein classes. This indicated that only 1.4% (29/1969) of the Ligand–Clusters and 6% (29/481) of the DBS supported the “one drug, one target” concept in the DBS4 dataset. The 39 promiscuous Ligand–Clusters were highly promiscuous—these interacted with 6.71 DBS on average. The high promiscuity of these 39 Ligand–Clusters can be explained by their ligands having to be rather small and adaptable to be able to interact with selective DBS pockets. This was not the case for the 29 selective Ligand–Clusters interacting with selective DBS. Subsequently, we then explored the pocket and ligand properties discriminating the 29 selective Ligand–Clusters from the 39 promiscuous Ligand–Clusters while using an ANOVA test. These two types of Ligand–Clusters tended to be small and flexible. However, the selective DBS pockets tended tend to have less tiny and more aliphatic residues, and to be more spherical. The corresponding ligands were very flexible and they presented a weak proportion of weak ratio between hydrogen donor and acceptor (HBD/HBA) and values of topological Polar Surface Area (tPSA). This suggests that these pockets can be more buried and more difficult to bind, and that only dedicated ligands with adapted properties can interact with these pockets. 

#### 2.3.3. Ligand–Cluster-DBS Interaction Network Examples

We studied two examples of interaction networks that integrate certain DBS and the Ligand–Clusters to which they interacted. 

Firstly, we visualized the 39 promiscuous Ligand–Clusters interacting with 71 selective DBS. These Ligand–Clusters additionally interacted with 142 promiscuous DBS (66 MP and 76 HP DBS) (Figure S5). This network resulted in 262 interactions between these 39 Ligand–Clusters and 213 DBS belonging to 14 different MOAD protein classes. The MOAD protein class and promiscuous level are indicated for each DBS. Some of these Ligand–Clusters are highly promiscuous. For instance, Ligand–Cluster numbers 1, 5, and 17 interacted with 27, 16, and 10 DBS, respectively, which belonged to 5, 7, and 4 different MOAD protein classes, respectively. Accordingly, the Ligand–Cluster numbers 1 and 5 assimilated to nucleotide derivatives or sugar, respectively, which are known to be well adapted to different binding sites. Ligand–Cluster number 17 clustered seven ligands, including triclosan (Drugbank ID: DB08604) and similar molecules. Triclosan is used as a preservative and antimicrobial agent in personal care products, cosmetics, kitchenware, toys, sports equipment, and footwear, and a review has reported that this molecule can have adverse effects on immune responses and cardiovascular functions [38]. Accordingly, we observed its high promiscuity and interaction with 10 DBS from four protein classes: oxidoreductases, transferases, transcription/translation, and transport proteins. These 39 mixed Ligand–Clusters interacted with both selective and promiscuous DBS. Finally, we observed 39 promiscuous Ligand–Clusters and 142 promiscuous DBS. This resulted in an interaction sub-network of 162 DBS sharing 24 Ligand–Clusters. This strong interconnection is mostly due to the 66% (142/213) of promiscuous DBS from different protein classes (Figure S5).

**Figure 7:**
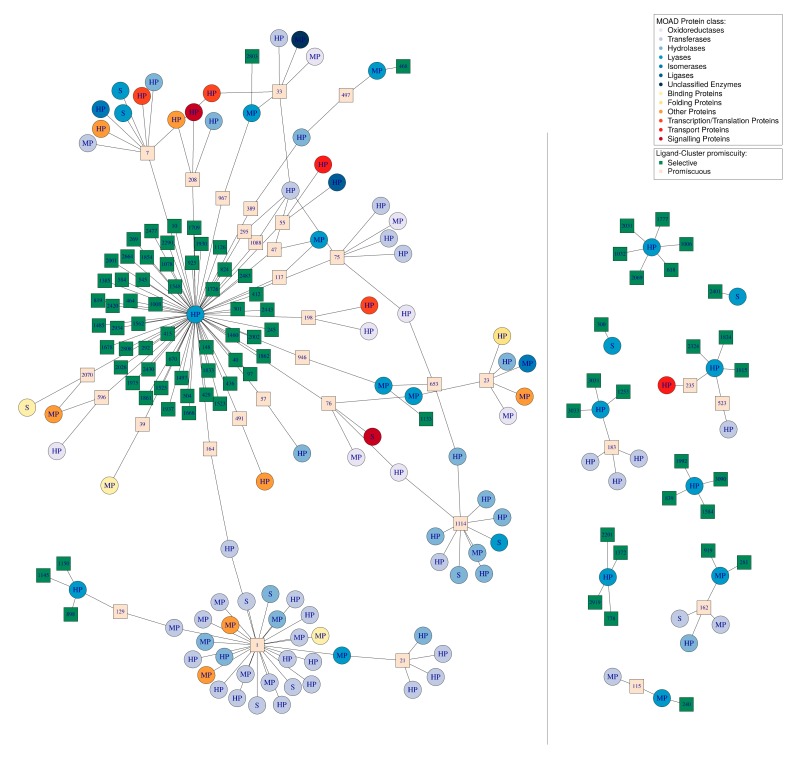
Network of the 20 DBS belonging to the lyase MOAD protein class in the DBS4 dataset, their 117 Ligand–Clusters, and the 84 other DBS from other protein classes that interact with these ligands, resulting in a total of 104 DBS and 220 interactions. Circles represent the DBS; these are colored according to the MOAD protein class to which they belong and are named according to their promiscuity (S for selective, MP for moderately promiscuous, and HP for highly promiscuous). The squares represent the Ligand–Clusters and are colored according to their level of promiscuity (beige for selective, binding to one DBS, or green for promiscuous) and their name is written inside the square. The grey line represents the boundary between the Pocket-Clusters and the Ligand–Clusters. This network visualization was created using the igraph package (see Section 4).

Secondly, we illustrated the impact of the DBS promiscuity by studying one protein class. We represented all of the Ligand–Clusters that interacted with the DBS from the lyase protein class and all of the DBS from other protein classes interacting with some of these Ligand–Clusters (Figure 7). This resulted in a network of 20 lyase DBS (five selective, eight MP, and seven HP DBS) and their 117 interacting Ligand–Clusters. These latter also interact with 84 DBS from 12 other protein classes, resulting in a total of 104 DBS and 220 interactions.

From the Ligand–Cluster point of view, we observed 85 (72%) dedicated and 32 (28%) promiscuous Ligand–Clusters according to the small portion of promiscuous Ligand–Clusters in the DBS4 dataset (Table 3). However, some of the promiscuous Ligand–Clusters were highly promiscuous, as illustrated by Ligand–Cluster number 1 in interaction with 27 DBS (Figure 7).

From the DBS point of view, only a small number (five) of the lyase DBS were not connected to another one DBS by some common Ligand–Clusters (two selective and three HP lyase DBS). Most of the lyase DBS shared at least one Ligand–Cluster with other DBS, and mainly with DBS from other protein classes. We observed one lyase DBS that was particularly promiscuous: DBS 1_1, corresponding to a human carbonic anhydrase protein, interacting with 73 Ligand–Clusters whose 55 were dedicated to this DBS. Mainly, we observed a high frequency of the promiscuous DBS (87.5% = 91/104) in this network. Consequently, a large sub-network, including most of the DBS (82.4%), were interconnected by at least one common Ligand–Cluster. This sub-network only included 27 promiscuous Ligand–Clusters, but 75 promiscuous DBS. This network illustrates the complexity of the interactions between DBS of different protein classes in terms of common Ligand–Cluster. It confirms that the high frequency of promiscuous DBS of different protein classes explained the interconnection in terms of Ligand–Cluster, even in this case of a weak frequency of promiscuous Ligand–Clusters.

## 3. Discussion

In this study, we used the high-quality protein-ligand complex database, the MOAD, to study the promiscuity of the druggable binding sites. MOAD PDB chains were clustered according to a sequence identity threshold of 90% in a homologous chain cluster using H-CD-HIT to identify DBS promiscuity by taking advantage of the increasing protein redundancy information. The high quality of the MOAD’s complexes is an advantage for characterizing binding site properties, but it can result in a limited number of available chains. Consequently, we studied the promiscuity of DBS bound to at least four drug-like valid ligands in the MOAD to reduce any false conclusions regarding selectivity due to the limited complex structures that are available. The resulting 481 DBS and 3,488 drug-like ligands, corresponding to 7,267 pocket-ligand interactions, confirmed that the MOAD can provide a valuable and large amount of data to study binding site promiscuity, for instance, through a comparison with datasets that Mestres et al. and Haupt et al. presented [2,9].

Our study highlights that promiscuous DBS are not an exceptional phenomenon, but they are common in most MOAD proteins. We mainly observed promiscuous DBS (80%) and a high frequency of HP DBS (45%) in interaction with at least four different Ligand–Clusters. This high occurrence of promiscuous DBS is in agreement with the findings by Gao and Skolnick (2013), who concluded that more than one-third of their representative pockets from the PDB were promiscuous and they interacted with multiple chemically different ligands [39]. However, these authors gathered 20,000 ligand-bound sites into only 1,000 representative pockets, which provided limited information regarding the promiscuity pocket characteristics. 

Using the high-precision MOAD allowed for the characterization of both the pockets and ligands that are associated with DBS of different promiscuities in terms of their geometrical and physicochemical properties. Specifically, we tested for the detectable tendencies of the pockets that are associated with DBS of different promiscuity, regardless of the protein class and their impact on the ability to interact with diverse ligands. We tested for the detectable tendencies of the ligands in interaction with DBS of different promiscuity. We found that 20% of the DBS were selective and their pockets were less favorable for establishing contact: they tended to be small, have a high proportion of side chain atoms, a low proportion of sulfur atoms and aliphatic residues, or had a few that were large, but weakly hydrophobic. Therefore, selective DBS interacted with a small portion of the Ligand–Cluster space (4%), which corresponded to small, highly adaptable, and hydrophilic ligands that presented low carbon and heavy atom proportions. As expected, few of these selective DBS exemplify the old concept of “one drug, one target”, as they belonged to nine different MOAD protein classes [40]. Complementarily, other selective DBS interacted with highly promiscuous Ligand–Clusters in interaction with, on average, nearly three MOAD protein classes. The high promiscuity of their Ligand–Clusters was in accordance with their ligand properties (high flexibility, small size, and presenting a particularly weak proportion of rotatable bonds), which not only allows them to interact with selective DBS, but also with various DBS.

The 45% of pockets that were associated with HP DBS mainly tended to be large and hydrophobic, or where a few of them were small, they presented high sulfur atom and aliphatic residue frequencies. These pocket properties were compatible with the flexibility of the DBS, which are known to contribute to promiscuity [22]. These pocket properties are expected to facilitate drug-like ligand interactions, as they are well adapted to bind to several and diverse ligands (their interacting Ligand–Clusters were associated, on average, with a Tanimoto coefficient of 0.44 (±0.10) per HP DBS). Accordingly, their ligands do not need a particular flexibility and they tended to be large with high carbon and heavy atom frequencies, and a few were hydrophilic.

The opposite promiscuity trend was observed in the MOAD for DBS and Ligand–Clusters: only 18% of Ligand–Clusters were promiscuous, whereas 80% of the DBS were promiscuous. The high frequency of selective Ligand–Clusters in the DBS4 dataset can be partly explained by the high frequency of ligands (24%) only observed once and by the clustering of homologous chains in one DBS, which thus reduces the number of DBS. However, this surprising opposite promiscuity tendency for DBS and Ligand–Clusters is in accordance with the analysis of compound and target promiscuity by Hu et al. [25]. These authors addressed the question of whether detectable tendencies for targets might exist to either recognize promiscuous or selective compounds, and how such tendencies might relate to the ability of targets to interact with increasing numbers of structurally diverse compounds. They concluded that the majority of compounds were only active against a single target, whereas most of the targets bound to varying numbers of promiscuous compounds. Next, they confirmed that less than 20% of their targets exclusively interacted with one selective compound, so the majority of the targets interacted with structurally diverse and promiscuous compounds [41]. This weak frequency of promiscuous Ligand that was observed both in our analysis and in a previous study [25] may be due to the fact that most of the ligands are not usually tested against other proteins beyond their target family, while proteins are usually tested against many different ligands, thus there is much more room for the observed promiscuity. Moreover, the known protein DBS universe is significantly smaller than the universe of possible ligand scaffolds. Rifaioglu et al. [42] recently underlined that there are tens of millions of compounds that are available in compound and bioactivity databases, (about 9,000 FDA-approved small molecule drugs approved by experimental), while there are roughly 550,000 reviewed protein records available (20,244 of which are human proteins) in protein sequence and annotations resources (e.g., UniProtKB/Swiss-Prot). Therefore, it could be quite expected that ligands are observed to be more selective.

We conclude the existence of a high, but variable, promiscuity of DBS related to the MOAD protein classes. This is in accordance with the conclusion by Mestres at al. [9] that the promiscuity depends on the protein families. We observed different promiscuity levels of DBS within different protein classes: numerous HP DBS and a few selective ones were present in most of samples. Finally, the analyses of the Ligand–Clusters and DBS interactions confirmed that DBS are highly interconnected by common Ligand–Clusters, regardless of the protein class to which they belong. Whereas, only 20% of the Ligand–Clusters were promiscuous, we conclude that these high interaction numbers between the DBS of different protein classes and Ligand–Clusters are a consequence of the high promiscuity of the DBS. The weak frequency of selective Ligand–Cluster could be due to the choice of a high quality but sparse database, such as MOAD. Nevertheless, this highlights that the high promiscuity of a large number of DBS compensates for a low number of promiscuous Ligand–Clusters. This analysis confirms that the DBS promiscuity strongly contributes to multiple interactions between Ligand–Clusters and DBS belonging to different protein classes. This is in accordance with Meyers et al. [14], who concluded that pocket similarity analysis only partly explains the drug promiscuity across different target families.

Computational approaches have been demonstrated to be very beneficial and promising for polypharmacological studies, but also drug off-target studies, whose mechanisms are poorly understood or largely unknown, in most cases [43]. Our high DBS promiscuity frequency results demonstrate the importance of simultaneously integrating the DBS and the ligand promiscuity information in protocols of multiple drug–target interactions. Taking into account the DBS promiscuity should contribute to explaining the drug promiscuity, the interfamily polypharmacology, presenting hypotheses for drug repositioning or off-target detection, accelerating drug development, and uncovering causes for adverse drug reactions.

## 4. Materials and Methods

### 4.1. MOAD Mining

The MOAD (Mother of All Databases) was used, as it is one of the largest databases that provides protein–ligand complexes. It corresponds to high-quality resolution structures that were extracted from the PDB (X-ray structures less than 2.5 Å): 25,769 high-resolution complexed structures were associated with 9,142 binding affinities [26,44]. 

#### 4.1.1. Drug-Like Ligand Space Analysis and Clustering

##### Ligand Selection

The MOAD includes a total of 12,440 different ligands. In this study, we focused on valid ligands defined as peptides of fewer than 11 amino acids, oligonucleotides of fewer than four nucleotides, and biologically relevant small molecules. The latter can include agonists, antagonists, cofactors, inhibitors, allosteric regulators, and enzymatic products, but it excludes covalently bonded molecules, crystallographic additives, salts, metals, and solvents.

We focused on drug-like ligands, as druggability is an important aspect of drug design [27]. Drug-like ligands correspond to orally bioavailable small drugs that have an optimal profile of physicochemical properties in terms of absorption, distribution, metabolism, excretion, and toxicity (ADME-Tox), as defined by Lipinski in 1997 [45] and reviewed by Perez-Nueno et al. in 2011 [46]. The FAF-Drugs3 server was used with the “drug-like soft” filter to select drug-like compatible ligands according to the ADME-Tox properties [47] and based on Lipinski’s rules, corresponding, for instance, to a Rings ≤ 6, a RotatableB ≤ 11, and the RatioH/C included between 0.1 and 1.11 values.

##### Drug-Like Ligand Clustering and Description

The diversity of this drug-like ligand set was analyzed while using Tanimoto similarity, based on MACCS fingerprints [48]. A Tanimoto coefficient that is equal to 1 corresponds to identical MACCS fingerprints, whereas a null coefficient does not share any fingerprint similarity. The Tanimoto coefficients were computed on all ligand pairs using OpenBabel software [49]. Similar ligands were gathered by hierarchical clustering while using the Butina algorithm [50] and Ward method aggregation criterion. Similar ligands with a Tanimoto coefficient threshold greater than or equal to 0.8, as in [29], were clustered and defined as a Ligand–Cluster.

The resulting ligands and Ligand–Clusters were described while using 21 geometrical and physicochemical descriptors that were proposed by FAF-Drugs3 software [47,51] to characterize their main tendencies relative to the promiscuity of their associated DBS. The ligand closest to the average properties of the ligands of each Ligand–Cluster in terms of weighted Euclidean distance was selected as the representative ligand.

#### 4.1.2. Protein Space Analysis and Clustering 

The MOAD proposes a protein classification in 18 disjoint classes: seven enzyme classes and 11 others, such as “Binding” (lectin, streptavidin, agglutinins, etc.); “Immune” (antibodies, immunoglobulins, cytokines, etc.); “Transport” (amino acid transporters, electron transport, etc.); and, “Structural” (actin, myosin, etc.). This was used as a reference, called the MOAD protein classes in our study, and corresponded to a large diversity of proteins and enzymes that was consistent for studying various protein classes in comparison to those of marketed small-molecule drug targets (MSMDT) [52].

Homologous protein chains were clustered in “homologous chain clusters” to quantify the different ligand partners of the considered binding sites in the MOAD.

**Figure 8:**
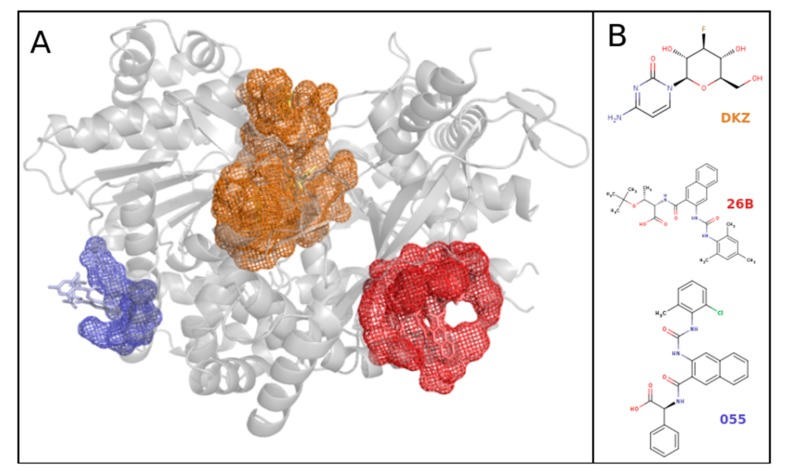
(**A**) Representation of the three pocket clusters (blue, orange, and red) observed on the 72 homologous protein chains from 67 PDB structures (five PDB structures had two chains each) of a family of transferase from *Homo sapiens*. These are illustrated on the PDB file as “glycogen phosphorylase from *Homo sapiens*” (PDB code: 3DDS) in grey. A total of 59 pockets were estimated on this homologous protein chain cluster: one pocket describes the blue binding site, four pockets describe the red binding site, and 54 pockets describe the orange site. (**B**) Representation of one representative ligand binding pocket (DZK, 26B, 055) from these three binding sites. The blue binding site fixed only one ligand, so it could not be determined in terms of promiscuity. The structure visualization was performed using PyMOL.

Each protein structure file may contain one or several chains. All of the mono-chains were hierarchically clustered based on their sequence identity while using the H-CD-HIT web server [29], which is a widely used sequence clustering tool for clustering that is based on sequence identity. A first clustering was performed to gather protein chains sharing more than 90% of sequence identity into families, and a second was performed on non-redundant family representatives sharing more than 80% of sequence identity. This two-step CD-HIT classification improves the protein-clustering quality, as it ensures that two similar chains (>90% sequence identity) are clustered into a common homologous chain cluster. Uddin et al. [53] used a sequence identity threshold of 80% to cluster orthologous proteins. Resulting homologous chain clusters may include none or several DBS. For instance, the cluster containing the *Homo sapiens* transferase encompasses a total of 72 mono-chains and three distinct DBS, as illustrated on protein glycogen phosphorylase from *Homo sapiens* (3DDS PDB code chain) in Figure 8. 

### 4.2. MOAD Druggable Binding Sites Extraction Protocol

Each DBS is described by a cluster of pockets estimated by proximity to valid drug-like ligands that are located at an identical region on superimposed homologous chains. DBS were extracted while using the following protocol: (1) we estimated all of the ligand-binding pockets through their proximity to valid drug-like ligands, (2) we superimposed homologous protein mono-chains, including pockets of each homologous chain cluster, and (3) we clustered pockets that were located at the same binding site into a Pocket-Cluster. Each Pocket-Cluster obtained from one homologous chain cluster corresponded to a DBS.

#### 4.2.1. Ligand-Binding Pocket Estimation 

The pockets are estimated by proximity to the co-crystallized ligand, as a set of atoms in close contact with a ligand. This close contact is defined by a given threshold to the ligand, commonly between 4 Å and 6 Å [54,55,56,57]. Here, the pockets were estimated by a proximity threshold of 5.5 Å, as used by Borrel et al. [34] using PockDrug-Server [33]. In this study, pockets that were located at the interface of several chains were omitted. Estimated pockets were characterized while using 72 physicochemical and geometrical descriptors, such as hydrophobicity, charge, atom, and residue composition described in [34], and the other 20 corresponded to the frequency of each residue to characterize the main tendencies of the pockets. 

#### 4.2.2. Superimposition of Mono-Chains from Each Homologous Chain Cluster 

All of the mono-chains within each homologous chain cluster were superimposed on the same reference to be able to locate the binding sites. TM-Align software [28] was used to generate the optimal superimposition of structures that are based on the structure similarity and sequence alignment.

#### 4.2.3. Cluster of Pockets Associated with a Druggable Binding Site 

A DBS was described by the set of overlapping pockets observed within the superimposed homologous chains, which are referred to as the Pocket-Cluster. It is known that binding sites can be difficult to precisely locate [34]. Here, a criterion was developed to cluster the overlapping pockets corresponding to one DBS. 

The steps of the Pocket-Clustering criterion are as follows, for each *p* pockets of one homologous chain cluster:

- let *n(i)* be the number of atoms of pocket *i*,
- let *g(i)* be the barycenter of the *n(i)* atoms of the pocket *i*,
- let d(i,j) be the Euclidean distance between *g(i)* and atom *j* of pocket *i* and $d_{max}\left(i\right)\;=\;\underset{j}{\max}d\left(i,j\right)$ the maximum value among the *n(i)* distances between the *n(i)* atoms and the barycenter *g(i)* of pocket *i*,
- let *D* and σ be the average value and standard deviation respectively of the population of the *p* observed values of $d_{max}\left(i\right)$ and
- the cutoff value based on *D* and σ from the *p* pockets from the homologous chain cluster used is (1):

(1) C= D−k×σ

where *k* is a constant parameter.

- Two pockets $i_{1}\;$ and $\;i_{2}$ are fall in the same Pocket-Cluster when $d\left(g(i_{1}\right),g(i_{2}))<C,$ meaning that the distance between $i_{1}$ barycenter and $i_{2}$ barycenter is lower than the cutoff value.

For our dataset, we experimentally found that *k* = 2 was an optimal value ensuring the transitivity property of our clustering criterion (i.e., when pockets $i_{1}$ and $i_{2}$ are in the same cluster and pockets $i_{2}$ and $i_{3}$ are in the same cluster, therefore $i_{1}$ and $i_{3}$ must be in the same cluster). Indeed, we do not obtain any non-overlapping pockets when we calculated the score of overlap (SO) between the *p* pockets of each DBS. It means that the disconnected pockets are clustered in two different Pocket-Clusters. Here, the score of overlap of [34,58] corresponds to the proportion of atoms that are common between two pockets that are associated with one binding site (2):

(2) $$SO=\;\frac{n_{common}}{n_{i_{1}}+n_{i_{2}}\;-\;n_{common}}$$

where *n*_common_ is the number of atoms belonging to both the pocket $\;i_{1\;}$ and pocket $\;i_{2}$, $n_{i_{1}}$ and $n_{i_{2}}$ are the numbers of atoms in pockets $\;i_{1}$ and $\;i_{2}$, respectively. The score of overlap values range from 0 to 1, and a score equal to 1 indicates the maximum overlap.

### 4.3. Promiscuity Characterization of Druggable Binding Site

#### 4.3.1. Determination of DBS Promiscuity

The promiscuity of a DBS is quantified by the number of different Ligand–Clusters in interaction with its Pocket-Cluster. Selective DBS was defined as DBS in interaction with only one Ligand–Cluster, similar to drug promiscuity and selectivity, as defined by Schneider et al. [59]. Promiscuous DBS was defined as DBS in interaction with more than one Ligand–Cluster. The higher the number of different Ligand–Clusters in interaction with a DBS, the higher the promiscuity of these DBS. Promiscuous DBS were split into two categories: moderately promiscuous DBS (MP DBS), which were those DBS in interactions with two or three Ligand–Clusters, and the highly promiscuous DBS (HP DBS), which were those observed in interactions with at least four Ligand–Clusters.

Only the DBS observed in more than one complexed chain (Pocket-Cluster size > 1) could be detected as promiscuous, and more than four complexed chains (Pocket-Cluster size ≥ 4) could be detected as HP in the MOAD.

#### 4.3.2. Analysis of DBS Promiscuity in Terms of Pocket and Ligand Properties

We studied the geometrical and physicochemical properties of the pockets that were associated with DBS of different promiscuities to highlight the trends explaining DBS promiscuity. We also studied the geometrical and physicochemical properties of the ligands to understand whether some of the properties explained their ability to interact with DBS of different promiscuity. We performed Student’s *t*-tests with a Bonferroni correction, ANOVA, and χ²-tests to compare the properties of the pockets or ligands that are associated with different DBS promiscuity.

We used Classification and Regression Trees (CART) to select a combination of descriptors that was able to discriminate pockets or ligands associated with selective versus HP DBS [60]. Model performance was evaluated using a five cross-validation approach to prevent overestimation [61]. We balanced the ratio between different selective DBS and HP DBS to train the CART due to the disequilibrium between occurrences of selective and HP pockets or ligands. For pocket prediction, all of the pockets from one Pocket-Cluster were assigned either to the training sample set or to the validation sample set to conduct the cross-validation. This avoided pockets that were associated with an identical DBS (expected to exhibit some similarity) in both the training and validation sets. The quality of these prediction models was evaluated by criteria, such as sensitivity (ability to predict true positive), specificity (ability to predict truly negative), and accuracy, as shown in Equations (3)–(5), respectively:

(3) $$Sensitivity\;=\frac{TP}{TP+FN}$$

(4) $$Specificity\;=\frac{TN}{TN+FP}$$

(5) $$Accucary\;=\frac{TP}{TP+FP}$$

where TP is true positive, TN is true negative, FP is false positive, and FN is false negative.

These analyses were performed using the “Stats” package (v3.6.0) implemented in R [62] and the library and function “rpart” in R [63].

### 4.4. Ligand–Cluster–DBS Interaction Network Illustration

We used a network approach through the igraph library of R software to visualize the interaction between DBS and Ligand–Clusters that are associated with DBS [64]. We drew some interactions between Ligand–Clusters and DBS in a network colored relative to the DBS promiscuity or MOAD protein classification. A DBS and a Ligand–Cluster was linked by an edge if one ligand of the Ligand–Clusters established an interaction with one pocket of the Pocket-Cluster that was associated with the considered DBS. Thus, these networks allowed for the visualization of the main interactions between the DBS and Ligand–Cluster.

## Figures and Tables

**Table 1:** The number (and proportion) of DBS in the DBS4 dataset according to their promiscuity level and correspondence with the number (and proportion) of binding pockets: selective (S, in interaction with one Ligand–Cluster), moderately promiscuous (MP, two or three Ligand–Clusters), and highly promiscuous (HP, four or more Ligand–Clusters).

	DBS		Pocket	
**S**	**100**	(20.8%)	791	(10.9%)
MP	166	(34.5%)	1447	(19.9%)
HP	215	(44.7%)	5029	(69.2%)
Total	481	(100.0%)	7267	(100.0%)

**Table 2:** Description of the Ligand–Clusters according to the different promiscuity levels using the DBS4 dataset. Ligands associated with selective (S), MP, and HP DBS are considered as dedicated, and those associated with DBS of different promiscuity levels are considered mixed. Second column gives the occurrence (and frequency) of Ligand–Clusters according to the promiscuity of DBS to which they bind: 29 were S, 182 were MP, the majority (1,631) were HP, and 127 were mixed, where they bind to DBS of different promiscuities. From the 127 mixed Ligand–Clusters, eight bound to selective and HP DBS; 26 bound to S, MP, and HP DBS; 88 bound to MP and HP DBS; and, five bound to selective and MP DBS. The third column is the average Tanimoto coefficient between the Ligand–Clusters. Ligands associated with S, MP, and HP were considered dedicated, and those associated with Pocket-Clusters with different promiscuity levels were considered as mixed. The last column is the number of corresponding ligands.

Ligand–Cluster	Occurrence (Frequency)			Tanimoto coefficient average (std. dev.)	Corresponding Ligands: Occurrence (Frequency)	
**Dedicated to**	**S DBS**	**29**	(1.5%)	0.24 (0.11)	53	(1.5%)
	MP DBS	182	(9.2%)	0.29 (0.12)	257	(7.4%)
	HP DBS	1631	(82.8%)	0.33 (0.13)	2621	(75.1%)
Mixed		127	(6.4%)	0.28 (0.13)	557	(16.0%)
ALL		1969	(100.0%)	0.33 (0.13)	3488	(100.0%)

**Table 3:** Distribution of the Ligand–Clusters relative to their ability to interact with DBS of different levels of promiscuity (rows). The last row, “All DBS”, indicates the values of all the Ligand–Clusters in the DBS4 dataset. The columns indicate the total number of Ligand–Clusters, and those that interacted with one DBS (selective) or more (promiscuous). For the promiscuous Ligand–Clusters, we detailed the average number of DBS bound and the average number of protein class bound (not indicated for the selective Ligand–Clusters, as they bind to one DBS).

Ligand–Cluster	Total	Selective	Promiscuous	Promiscuous	Promiscuous
	Occurrence	Occurrence	Occurrence	Number of DBS: Average (sd)	Number of Protein Class: Average (sd)
Selective DBS	68	29	39	6.7 (5.37)	2.9 (1.45)
MP DBS	301	168	133	4.2 (3.68)	2.1 (1.32)
HP DBS	1753	1421	332	3.2 (2.60)	1.8 (1.02)
All DBS^1^	1969	1618	351	3.1 (2.54)	1.8 (1.02)

^1^ The row “All DBS” is not the sum of the other rows because some Ligand–Cluster bind several types of DBS

## Supplementary Materials

The following are available online. Table S1: ANOVA of selective, MP and HP DBS pockets, Table S2: ANOVA of Ligand–Clusters associated to selective, MP and HP DBS, Table S3: ANOVA of Ligand–Clusters associated to selective DBS and Mixed Ligand–Clusters, Table S4: Repartition of DBS among the 17 MOAD protein classes, Figure S5: Network of 39 promiscuous Ligand–Clusters.

## Abbreviations

Druggable Binding Site (DBS), Selective (S), Moderately Promiscuous (MP), Highly Promiscuous (HP), Mother Of All Databases (MOAD), Classification And Regression Trees (CART), 2 dimensions (2D), 3 Dimensions (3D), Protein Data Bank (PDB), Marketed Small-Molecule Drug Targets (MSMDT); Cyclin-dependent kinase (CDK); Pocket Convexity Index (PCI)
