# Supplementary material for: High Impact: The Role of Promiscuous Binding Sites in Polypharmacology
Source: Molecules. 2019 Jul 10;24(14):2529. doi: 10.3390/molecules24142529 (PMC6680532; doi:10.3390/molecules24142529)

Table S1. All the pocket descriptors (except the frequency of each atoms) sorted according to the average comparison test (ANOVA non parametric) between the different degree of promiscuity. The *p*-value range is: $<1x10^{-20}$ (***), $<1x10^{-10}$ (**), $<5x10^{-2}$ (*) and $>5x10^{-2}$(-). The threshold of significance has been determined with the Bonferroni correction (alpha risk divided by the number of tests). Other columns are the average and the standard deviation of the 791, 1447, 5029 and 7267 pockets from the selective, MP, HP and all the DBS from the DBS4 dataset.

Pocket Descriptor	*p*-value	S DBS		MP DBS		HP DBS		Total	
		Av[1]	Sd[2]	Av	Sd	Av	Sd	Av	Sd
SURFACE_HULL	***	538.3	144.1	596.5	175.9	679.7	166.6	647.7	173.6
RADIUS_HULL	***	8.66	1.31	9.11	1.48	9.84	1.47	9.57	1.52
DIAMETER_HULL	***	17.14	2.68	18.00	2.97	19.48	2.97	18.93	3.06
VOLUME_HULL	***	1031.6	408.5	1214.6	538.3	1459.1	530.6	1363.9	541.6
RADIUS_CYLINDER	***	8.48	1.35	8.90	1.49	9.62	1.49	9.35	1.54
SMALLEST_SIZE	***	9.76	1.67	10.32	1.96	11.03	1.66	10.75	1.78
C_res	***	16.62	4.30	18.14	5.34	20.12	5.10	19.34	5.21
C_ATOM	***	72.30	23.08	79.79	28.23	91.02	29.46	86.75	29.36
FACE	***	61.30	13.85	65.43	14.81	70.62	14.42	68.57	14.80
p_main_chain_atom	***	0.328	0.145	0.313	0.140	0.375	0.136	0.358	0.140
p_side_chain_atom	***	0.672	0.145	0.687	0.140	0.625	0.136	0.642	0.140
X._ATOM_CONVEXE	***	0.470	0.079	0.457	0.080	0.430	0.079	0.440	0.081
p_sulfur_atom	***	0.006	0.010	0.009	0.012	0.013	0.014	0.011	0.013
p_O_atom	***	0.076	0.035	0.071	0.035	0.086	0.031	0.082	0.033
hydrophobic_kyte	***	-0.158	1.021	-0.248	1.121	0.182	0.960	0.060	1.018
p_Ntrp_atom	***	0.004	0.009	0.007	0.011	0.003	0.007	0.004	0.009
polarity	***	0.146	0.084	0.138	0.077	0.171	0.079	0.161	0.080
p_positive_res	***	0.122	0.094	0.137	0.093	0.104	0.075	0.112	0.082
p_polar_res	***	0.536	0.174	0.549	0.178	0.487	0.162	0.504	0.169
p_charged_res	***	0.224	0.135	0.255	0.148	0.208	0.112	0.219	0.124
p_aliphatic_res	***	0.206	0.122	0.194	0.124	0.237	0.126	0.225	0.127
p_Car_atom	***	0.200	0.111	0.209	0.129	0.170	0.118	0.181	0.121
p_hydrophobic_res	***	0.689	0.133	0.692	0.158	0.729	0.117	0.717	0.129
p_ND1_atom	***	0.009	0.013	0.011	0.015	0.007	0.011	0.008	0.012
p_S_atom	***	0.002	0.005	0.002	0.006	0.004	0.008	0.003	0.007
p_NE2_atom	***	0.010	0.014	0.012	0.016	0.008	0.012	0.009	0.013
p_nitrogen_atom	***	0.146	0.049	0.147	0.046	0.135	0.041	0.138	0.043
p_aromatic_res	***	0.232	0.119	0.257	0.153	0.218	0.128	0.227	0.134
p_Ocoo_atom	***	0.006	0.009	0.009	0.013	0.007	0.010	0.007	0.011
p_Cgln_atom	**	0.012	0.013	0.011	0.014	0.009	0.010	0.009	0.011
p_N_atom	**	0.092	0.042	0.091	0.038	0.100	0.039	0.097	0.039
hydrophobicity	**	0.033	0.031	0.030	0.031	0.038	0.028	0.036	0.029
INERTIA_2	**	0.308	0.054	0.301	0.045	0.293	0.049	0.296	0.049
charge	**	0.314	1.738	0.431	2.134	-0.012	1.826	0.111	1.892
p_Ccoo_atom	**	0.016	0.015	0.018	0.019	0.015	0.013	0.015	0.015
p_tiny_res	**	0.183	0.103	0.177	0.117	0.199	0.111	0.193	0.111
p_negative_res	*	0.102	0.079	0.117	0.111	0.104	0.072	0.106	0.082
p_Nlys_atom	*	0.004	0.007	0.005	0.010	0.005	0.007	0.005	0.008
p_C_atom	*	0.166	0.065	0.155	0.058	0.164	0.057	0.163	0.059
p_hyd_atom	*	0.113	0.043	0.120	0.045	0.113	0.044	0.115	0.044
p_oxygen_atom	*	0.159	0.044	0.156	0.046	0.153	0.038	0.154	0.041
p_Otyr_atom	*	0.014	0.014	0.013	0.014	0.012	0.013	0.012	0.013

INERTIA_1	*	0.511	0.077	0.513	0.068	0.521	0.070	0.518	0.071
p_hydrophobic_atom	*	0.122	0.042	0.126	0.045	0.121	0.044	0.122	0.044
p_carbone_atom	*	0.676	0.084	0.681	0.073	0.686	0.084	0.684	0.082
PCI	*	0.025	0.012	0.027	0.013	0.026	0.012	0.026	0.012
INERTIA_3	*	0.181	0.048	0.186	0.047	0.186	0.041	0.185	0.043
CONV.SH_COEFF	*	0.978	0.025	0.976	0.028	0.978	0.026	0.978	0.027
p_Carg_atom	*	0.017	0.015	0.016	0.015	0.016	0.017	0.016	0.016
p_Ooh_atom	*	0.017	0.015	0.016	0.015	0.016	0.017	0.016	0.016
PSI[3]	-	0.541	0.083	0.541	0.079	0.537	0.066	0.538	0.071
p_small_res[3]	-	0.437	0.132	0.423	0.137	0.428	0.143	0.428	0.141

[1] Average

[2] Standard deviation

[3] These descriptors are not significant (*p*-value>5x10^{-2})

Table S2. All the ligand descriptors sorted according to the average comparison test (ANOVA non parametric) between the different degree of promiscuity. The *p*-value range is: <1x10^{-10} (***), <1x10^{-5} (**), <0.01 (*) and >0.01(-). The threshold of significance has been determined with the Bonferroni correction (alpha risk divided by the number of tests). Other columns are the average and the standard deviation of the 53, 257, 2621 and 3488 ligands from the Ligand-Clusters that respectively bind to selective, MP, HP and all the DBS from the DBS4 dataset.

**Ligand Descriptor**	***p*-value**	**S DBS**		**MP DBS**		**HP DBS**		**Total**	
		Av[1]	Sd[2]	Av	Sd	Av	Sd	Av	Sd
Rings	***	1.11	0.58	1.91	1.10	2.27	0.98	2.22	1.00
RigidB	***	10.57	5.83	15.27	7.38	18.12	6.70	17.74	6.87
HeavyAtoms	***	16.43	5.70	21.17	8.09	23.49	7.46	23.16	7.57
CarbonAtoms	***	11.47	4.00	15.12	6.52	16.82	5.89	16.57	5.98
logSw	***	-1.91	1.63	-2.90	1.70	-3.40	1.46	-3.33	1.50
MW	***	241.37	86.35	309.06	115.39	337.56	107.03	333.32	108.44
Solubility (mg/l)	***	8.2x10^{4}	1.0x10^{5}	4.6x10^{4}	9.4x10^{4}	2.5x10^{4}	5.9x10^{4}	2.8x10^{4}	6.4x10^{4}
NumCharges	***	1.00	0.94	0.77	0.73	0.54	0.71	0.57	0.72
logD	**	0.05	2.49	0.49	2.56	1.40	2.22	1.29	2.27
HBA	**	4.00	2.07	5.25	2.06	5.60	2.21	5.54	2.20
HeteroAtoms	**	4.96	3.09	6.05	2.36	6.67	2.62	6.59	2.62
TotalCharge	**	-0.17	0.80	-0.27	0.80	0.00	0.73	-0.03	0.74
logP	**	1.09	2.19	1.94	1.92	2.42	1.69	2.35	1.74
HBD/HBA	**	5.81	3.08	7.55	2.78	7.93	2.99	7.85	2.99
MaxSizeRing	**	7.26	3.79	7.12	2.92	7.98	2.69	7.89	2.75
Flexibility	*	0.30	0.23	0.23	0.16	0.20	0.13	0.20	0.13
tPSA	*	70.71	42.16	88.54	32.71	89.64	34.60	89.20	34.67
HBD	*	1.81	1.29	2.31	1.25	2.33	1.32	2.32	1.31
RotatableB	*	3.74	2.53	4.04	2.40	4.37	2.53	4.33	2.53
Lipinski Violation[3]	-	0.02	0.14	0.11	0.35	0.12	0.38	0.12	0.37
ratioH/C[3]	-	0.46	0.25	0.45	0.19	0.43	0.18	0.43	0.19

[1] Average

[2] Standard deviation

[3] These descriptors are not significant (*p*-value>0.01)

Table S3. All the ligands descriptors sorted according to the average comparison test (non-parametric ANOVA) between the 53 ligands from the 29 Ligand-Clusters that bind to selective DBS and the 250 ligands from the 39 Mixed Ligand-Clusters that bind to at least one selective DBS. The *p*-value range is: $<1x10^{-5}$ (**), $<5x10^{-2}$(*) and $>5x10^{-2}$(-). The threshold of significance has been determined with the Bonferroni correction (alpha risk divided by the number of tests). Other columns are the average and the standard deviation of each class of ligands.

**Ligand Descriptor**	***p*-value**	**Selective DBS**		**Mixed**	
		Av[1]	std.dev [2]	Av	Std.dev
HBD	**	1.81	1.29	3.11	1.49
HBD/HBA	**	5.81	3.08	8.70	3.60
HBA	**	4.00	2.07	5.59	2.51
Rings	*	1.11	0.58	1.47	0.76
tPSA	*	70.71	42.16	94.28	36.27
NumCharges	*	1.00	0.94	0.51	0.70
ratioH/C	*	0.46	0.25	0.59	0.30
Flexibility	*	0.30	0.23	0.20	0.17
RigidB	*	10.57	5.83	12.72	6.07
HeteroAtoms	*	4.96	3.09	5.98	2.53
RotatableB	*	3.74	2.53	2.93	2.56
HeavyAtoms	*	16.43	5.70	18.23	6.26
Lipinski.Violation[3]	-	0.02	0.14	0.07	0.27
logD[3]	-	0.05	2.49	-0.66	2.72
MW[3]	-	241.37	86.35	264.92	87.92
logP[3]	-	1.09	2.19	0.52	2.38
Solubility (mg/l)[3]	-	$8.23x10^4$	$1.03x10^4$	$1.08x10^5$	$1.17x10^5$
CarbonAtoms[3]	-	11.47	4.00	12.24	5.77
logSw[3]	-	-1.91	1.63	-1.75	1.79
MaxSizeRing[3]	-	7.26	3.79	7.63	3.89
TotalCharge[3]	-	-0.17	0.80	-0.22	0.73

[1] Average

[2] Standard deviation

[3] These descriptors are not significant (*p*-value>0.01)

Table S4. Repartition of the 481 DBS among the 17 MOAD protein classes represented at least once in our DBS4 dataset. The columns respectively represent the number of DBS per MOAD protein class, their proportion, the average number of pocket per DBS, the number of selective, MP and HP DBS, the number of Ligand-Clusters and the average promiscuity per DBS for each class.

MOAD Protein Class	Occurrence	Prop. of class	Average number of pocket/DBS	S	MP	HP	Number of Ligand-Cluster	Average prom.
Transferases	134	27.86	15.38	18	36	80	741	5.5
Hydrolases	126	26.2	15.02	26	37	63	602	4.8
Oxidoreductases	86	17.88	13.49	22	40	24	243	2.8
Other Prot.	24	4.99	11.21	7	13	4	92	3.8
Lyases	20	4.16	16.50	5	8	7	117	5.9
Binding Prot.	20	4.16	11.30	9	10	1	24	1.2
Isomerases	15	3.12	10.07	3	8	4	46	3.1
Transcription/ Translation Prot.	15	3.12	23.53	2	3	10	97	6.5
Signaling Prot.	13	2.7	23.38	1	4	8	69	5.3
Ligases	8	1.66	14.13	3	0	5	39	4.9
Transport Prot.	7	1.46	16.43	0	3	4	41	5.9
Unclassified Enzymes	4	0.83	9.00	1	2	1	9	2.3
Folding Prot.	3	0.62	57.67	0	1	2	94	31.3
Immune Prot.	2	0.42	5.50	1	0	1	5	2.5
Mobility Prot.	2	0.42	20.00	1	0	1	12	6.0
Cell Cycle Prot.	1	0.21	6.00	0	1	0	2	2.0
Structural Prot.	1	0.21	27.00	1	0	0	1	1.0

Figure S5. Network of the 39 promiscuous Ligand-Clusters in interaction with both selective and promiscuous DBS, composed of 213 DBS resulting in 262 interactions. The circles represent the 213 DBS; they are colored according to the MOAD protein class which they belong and named according to their promiscuity. The squares represent the 39 mixed Ligand-Clusters, with their number written inside. The grey lines represent the interactions between DBS and Ligand-Clusters. This network visualization is made by the igraph package.

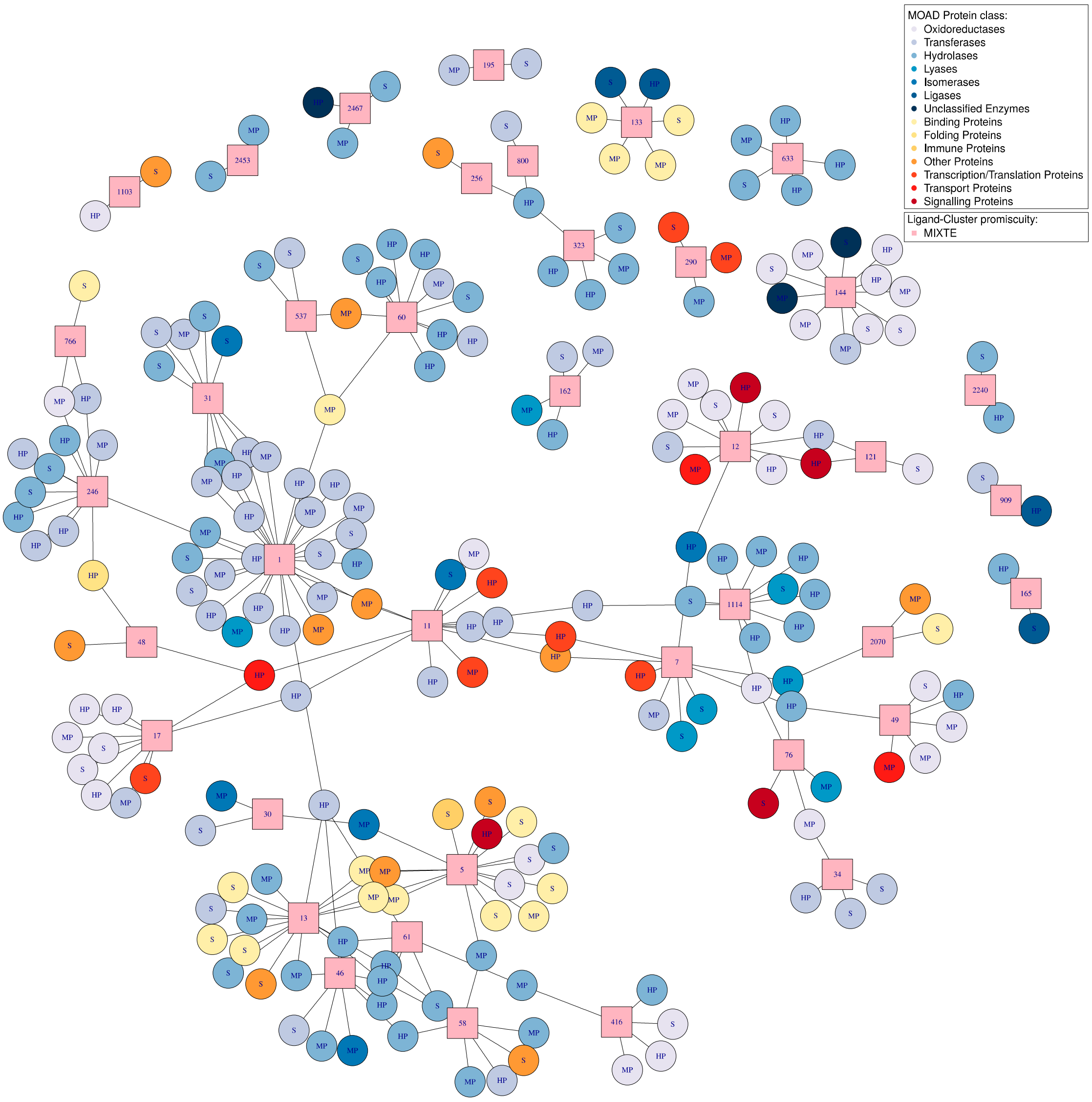

Supplement: Supplementary file 1 [file molecules-24-02529-s001.pdf]
